# Supplementary material for: Structural Basis and Mode of Action for Two Broadly Neutralizing Antibodies Against SARS-CoV-2 Emerging Variants of Concern
Source: bioRxiv. 2021 Aug 3:2021.08.02.454546. Preprint. [Version 1] doi: 10.1101/2021.08.02.454546 (PMC8351775; doi:10.1101/2021.08.02.454546)
Supplement: 1 — Figure S1. CV3–1 and CV3–25 Neutralize SARS-CoV-2 Variants In Vitro and Protect In Vivo. Related toFigure 1. (A, B) Cell-surface staining of 293T cells expressing full-length Spike from indicated variants (B.1.1.7, B.1.351, P.1, B.1.429, B.1.526, B.1.525) or their corresponding individual mutations by CV3–1 (A) and CV3–25 (B) mAbs. The graphs show median fluorescence intensities (MFIs). Dashed lines indicate the reference value obtained with Spike D614G. Error bars indicate means ± SEM. These results were obtained in at least two independent experiments. Statistical significance was tested using Kruskal-Wallis test with a Dunn’s post-test (*p < 0.05; ns, non significant). (C) Viral loads (FFUs/mg) from indicated tissue using Vero E6 cells as targets in mice prophylactically treated with CV3–1 and CV3–25 GASDALIE for the experiment shown in Figure 1C. Undetectable virus amounts were set to 1. (D) A plot showing mRNA levels SARS-CoV-2 nucleocapsid (N gene) from nose, lung and brain tissues of K18-hACE2 mice after sacrifice at times indicated in Figure1E. (E-F) A plot showing mRNA levels of indicated cytokines from lung and brain tissues of K18-hACE2 mice after sacrifice at times indicated in Figure1E. The mRNA amounts in (D-F) were normalized to Gapdh mRNA and to levels seen in uninfected mice. Viral loads and inflammatory cytokine profile in indicated tissues were determined after necropsy for mice that succumb to infection at day 6 and for surviving mice at 10 dpi. Grouped data in (C-F) were analyzed by 2-way ANOVA followed by Tukey’s multiple comparison tests. Figure S2. Conformational Dynamics of CV3–1 and CV3–25 Bound SB.1.1.7. Related toFigure 2. (A-C) Tilt angles of spikes on unliganded, CV3–1 Fab treated, and CV3–25 Fab treated pseudoviruses. Scheme graph of tilt angle is shown in (E). (D) The binding of CV3–1 or CV3–25 to SARS-CoV-2 S D614G expressed on 293T cells was measured flow cytometry. Cells were incubated with increasing amounts of mAbs and their bind [file NIHPP2021.08.02.454546V1-supplement-1.pdf]

bioRxiv preprint doi: https://doi.org/10.1101/2021.08.02.454546; this version posted August 3, 2021. The copyright holder for this preprint (which was not certified by peer review) is the author/funder. This article is a US Government work. It is not subject to copyright under 17 USC 105 and is also made available for use under a CC0 license.

Table S1. CryoET Data Acquisition and Image Processing

**Sample**	**CV3-1**	**CV3-25**	**unliganded**
**Data Collection**			
Microscope	FEI Titan Krios	FEI Titan Krios	FEI Titan Krios
Voltage (kV)	300	300	300
Energy-filter (ev)	20	20	20
Detector	Gatan K3	Gatan K3	Gatan K3
Recording Mode	Counting	Counting	Counting
Pixel size (Å)	1.346	1.346	1.346
Defocus range (µm)	-2 to -5	-2 to -5	-2 to -5
Acquisition scheme	-60°/60 °, 3 °	-60°/60 °, 3 °	-60°/60 °, 3 °
Total Dose (e/Å^2)	~120	~120	~120
Frame number	10	10	10
Tomograms	56	49	63
**Image processing**			
Virus particles	298	299	345
Subtomograms	1353	7739	9967
Symmetry	C3	C1	C1

bioRxiv preprint doi: https://doi.org/10.1101/2021.08.02.454546; this version posted August 3, 2021. The copyright holder for this preprint (which was not certified by peer review) is the author/funder. This article is a US Government work. It is not subject to copyright under 17 USC 105 and is also made available for use under a CC0 license.

Resolution at 0.143 FSC (Å)	12	11	10
EMDB ID			

Table S2. Crystallographic Data Dollection and Refinement Statistics.

	**CV3-25 Fab_ $S2_{1140-1165}$ peptide complex**
**Data collection**	
Wavelength, Å	0.979
Resolution range, Å	38.6 - 2.15 (2.23 - 2.15)
Space group	$P2_1$
Unit cell parameter	
a, b, c, Å	82.8, 85.2, 87.1
α, β, γ, °	90, 114.77, 90
Redundancy	25.7 (2.0)
Completeness, %	96.5 (81.9)
Mean I/sigma(I)	6.18 (2.3)
R_{merge}[a]	0.185 (0.489)
R_{pim}[b]	0.119 (0.329)
$CC_{1/2}$[c]	0.942 (0.714)
Wilson B_{factor}, ($1/Å^2$)[d]	38.7
**Refinement**	
R_{work}[e]	0.196 (0.256)
R_{free}[f]	0.239 (0.290)
Resolution, Å	38.6 - 2.15
# of non-hydrogen atoms	
proteins	7,032
water	530
Overall B_{factor}, ($Å^2$)	
proteins	45
ligands	58
water	47
RMS (bond lengths), Å	0.009
RMS (bond angles), °	1.17
Ramachandran[g]	
Favored, %	97.8

bioRxiv preprint doi: https://doi.org/10.1101/2021.08.02.454546; this version posted August 3, 2021. The copyright holder for this preprint (which was not certified by peer review) is the author/funder. This article is a US Government work. It is not subject to copyright under 17 USC 105 and is also made available for use under a CC0 license.

Allowed, %	2.2
Outliers, %	0.0
PDB ID	7NAB

Statistics for the highest-resolution shell are shown in parentheses.

[a]$R_{merge} = \sum | I - | / \sum I$, *where I is the observed intensity and is the average intensity obtained from multiple observations of symmetry-related reflections after rejections*

[b]R_{pim} *= as defined in (Weiss, 2001)*

[c]$CC_{1/2}$ *= as defined by Karplus and Diederichs (Karplus and Diederichs, 2012)*

[d]*Wilson* B_{factor} *as calculated in (Popov and Bourenkov, 2003)*

[e]$R = \sum \| F_o | - | F_c \| / \sum | F_o |$, *where* F_o *and* F_c *are the observed and calculated structure factors, respectively*

[f]R_{free} *= as defined by Brünger (Brunger, 1997)*

[g]*Calculated with MolProbity*

Table S3. Cryo-EM Data Collection and Refinement Statistics

Protein	***CV3-25 Fab_ SARS-CoV-2 HexaPro spike complex***
EMDB	*TBD*
<u>Data collection and Reconstruction</u>	
Microscope	*Titan Krios*
Voltage (kV)	*300*
Electron dose (e⁻/Å)	*59.3*
Detector	*K2-summit with Bioquantum Image Filter*
Magnification	*165,000*
Pixel size (Å/pixel)	*0.821*
Defocus range (µm)	*0.537 – 2.351*
Micrographs collected	*6460*

bioRxiv preprint doi: https://doi.org/10.1101/2021.08.02.454546; this version posted August 3, 2021. The copyright holder for this preprint (which was not certified by peer review) is the author/funder. This article is a US Government work. It is not subject to copyright under 17 USC 105 and is also made available for use under a CC0 license.

Particles extracted/final	*1630360 / 184874*
Symmetry imposed	*C1*
Box size (pixel)	*432*
Unmasked and masked resolution at 0.143 FSC (Å)	*4.09 / 3.49*

Figure S1

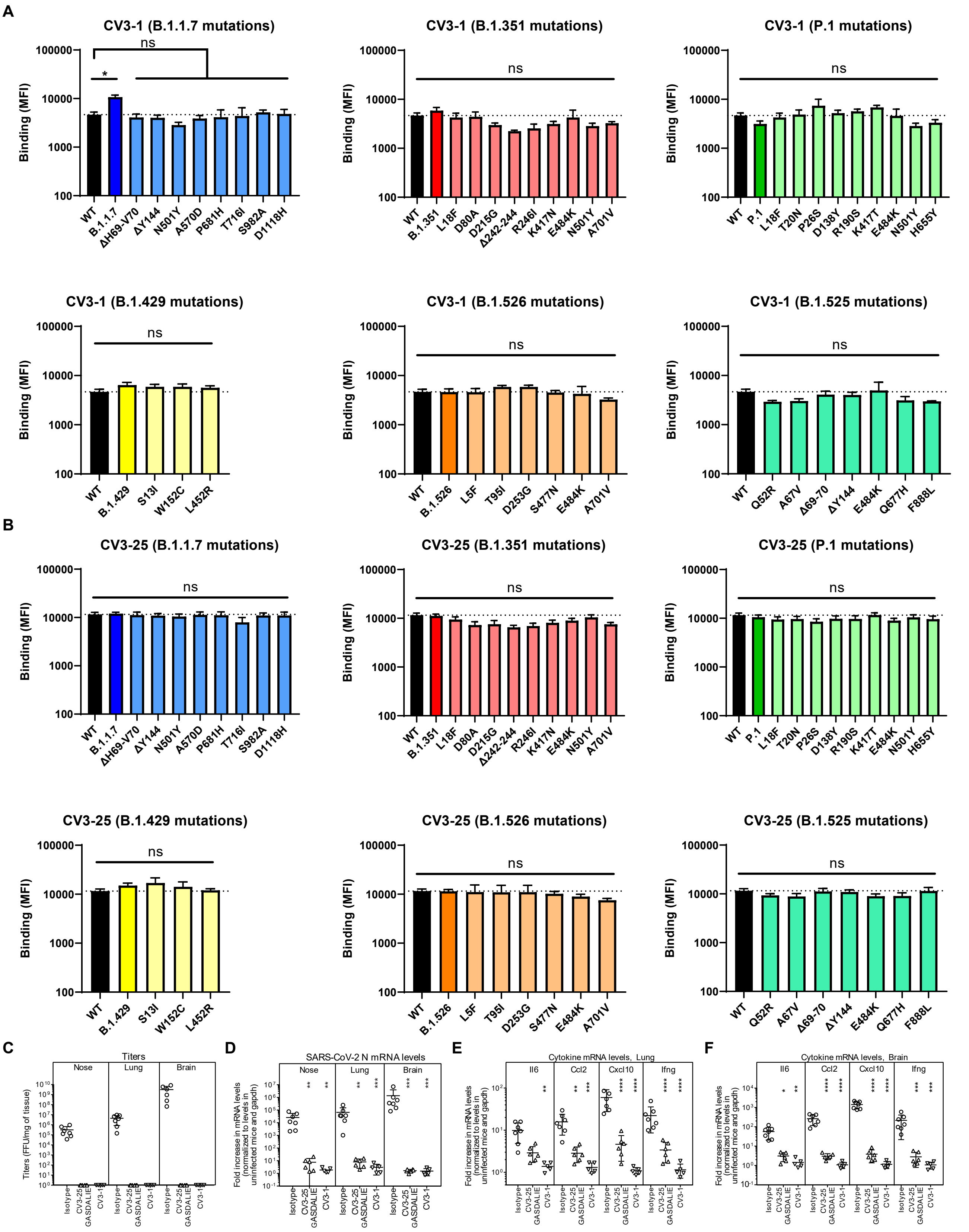
A
CV3-1 (B.1.1.7 mutations)
ns
*
Binding (MFI)
100000
10000
1000
100
WT
B.1.1.7
ΔH69-V70
ΔY144
N501Y
A570D
P681H
T716I
S982A
D1118H
CV3-1 (B.1.351 mutations)
ns
WT
B.1.351
L18F
D80A
D215G
Δ242-244
R246I
K417N
E484K
N501Y
A701V
CV3-1 (P.1 mutations)
ns
WT
P.1
L18F
T20N
P26S
D138Y
R190S
K417T
E484K
N501Y
H655Y
CV3-1 (B.1.429 mutations)
ns
WT
B.1.429
S13I
W152C
L452R
CV3-1 (B.1.526 mutations)
ns
WT
B.1.526
L5F
T95I
D253G
S477N
E484K
A701V
CV3-1 (B.1.525 mutations)
ns
WT
Q52R
A67V
Δ69-70
ΔY144
E484K
Q677H
F888L
B
CV3-25 (B.1.1.7 mutations)
CV3-25 (B.1.351 mutations)
CV3-25 (P.1 mutations)
CV3-25 (B.1.429 mutations)
CV3-25 (B.1.526 mutations)
CV3-25 (B.1.525 mutations)
C
Titers
Titers (FFU/mg of tissue)
Nose
Lung
Brain
Isotype
CV3-25 GASDALIE
CV3-1
D
SARS-CoV-2 N mRNA levels
Fold increase in mRNA levels (normalized to levels in uninfected mice and gapdh)
Nose
Lung
Brain
E
Cytokine mRNA levels, Lung
Il6
Ccl2
Cxcl10
Ifng
F
Cytokine mRNA levels, Brain
Il6
Ccl2
Cxcl10
Ifng

Figure S2

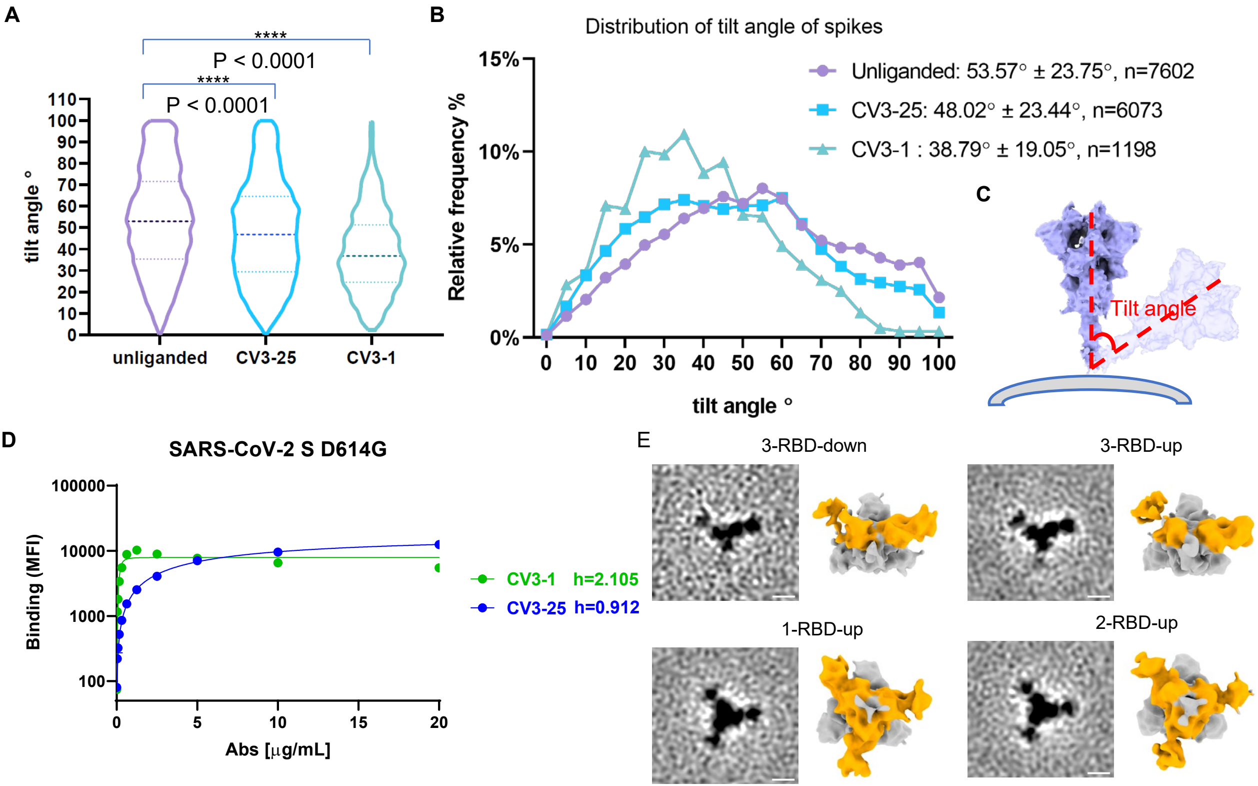

A
****
P < 0.0001
****
P < 0.0001
tilt angle °
unliganded
CV3-25
CV3-1
B
Distribution of tilt angle of spikes
Relative frequency %
Unliganded: 53.57° ± 23.75°, n=7602
CV3-25: 48.02° ± 23.44°, n=6073
CV3-1 : 38.79° ± 19.05°, n=1198
tilt angle °
C
Tilt angle
D
SARS-CoV-2 S D614G
Binding (MFI)
Abs [μg/mL]
CV3-1 h=2.105
CV3-25 h=0.912
E
3-RBD-down
3-RBD-up
1-RBD-up
2-RBD-up

Figure S3

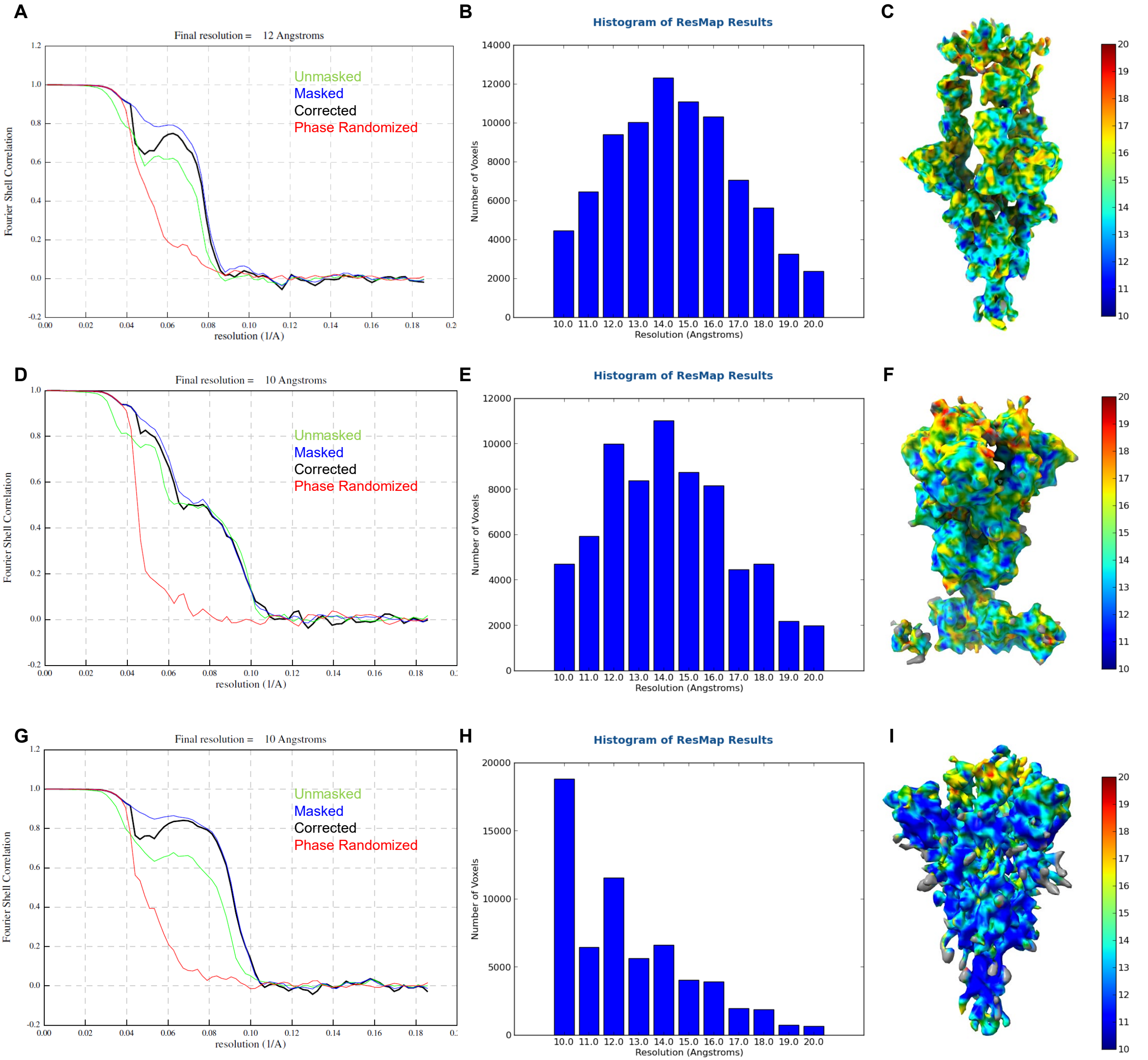

A
Final resolution = 12 Angstroms
Unmasked
Masked
Corrected
Phase Randomized
Fourier Shell Correlation
resolution (1/A)
B
Histogram of ResMap Results
Number of Voxels
Resolution (Angstroms)
C
D
Final resolution = 10 Angstroms
Unmasked
Masked
Corrected
Phase Randomized
Fourier Shell Correlation
resolution (1/A)
E
Histogram of ResMap Results
Number of Voxels
Resolution (Angstroms)
F
G
Final resolution = 10 Angstroms
Unmasked
Masked
Corrected
Phase Randomized
Fourier Shell Correlation
resolution (1/A)
H
Histogram of ResMap Results
Number of Voxels
Resolution (Angstroms)
I

Figure S4

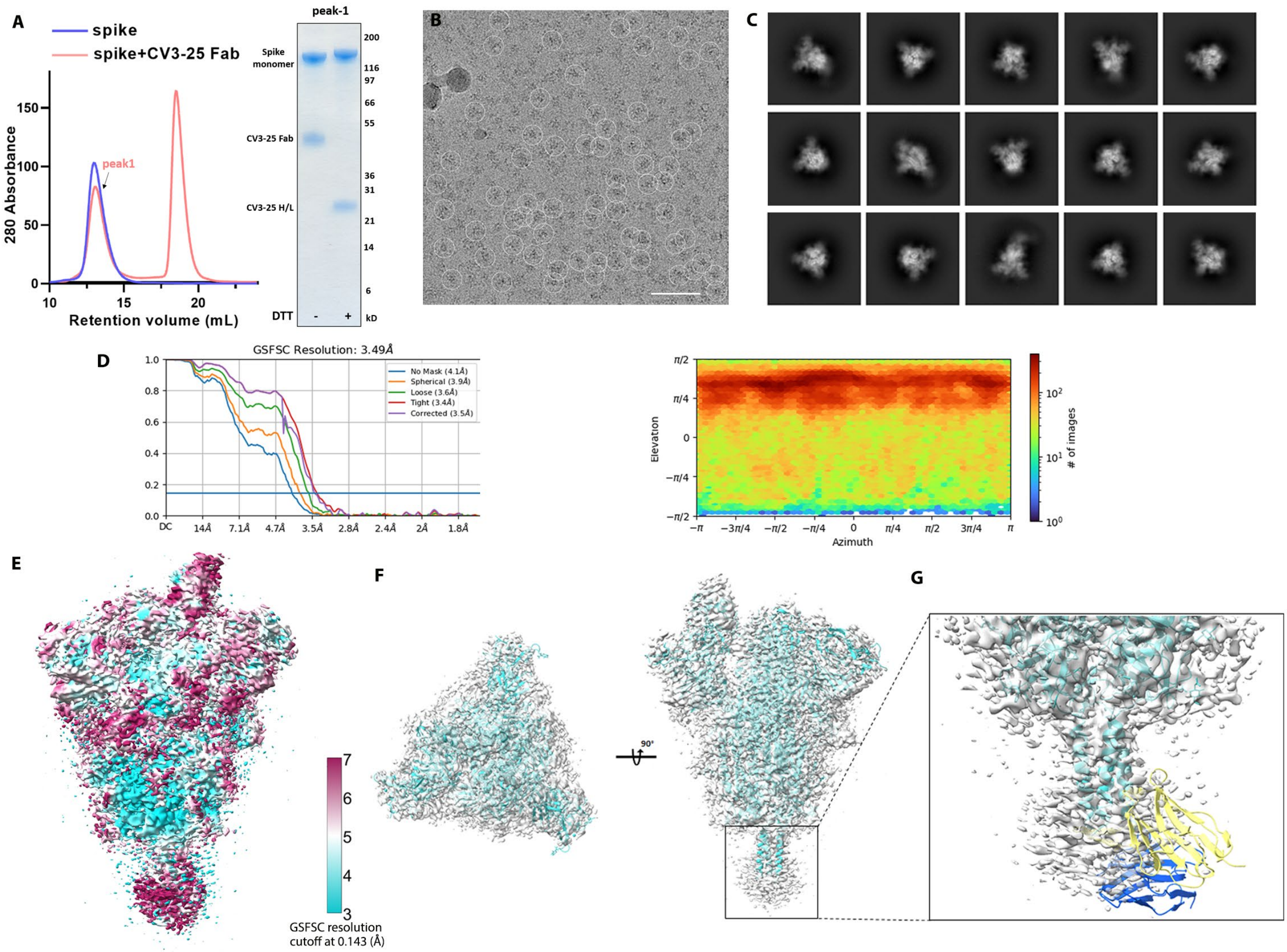

A
spike
spike+CV3-25 Fab
peak1
280 Absorbance
Retention volume (mL)
peak-1
Spike monomer
CV3-25 Fab
CV3-25 H/L
DTT
kD
B
C
D
GSFSC Resolution: 3.49Å
No Mask (4.1Å)
Spherical (3.9Å)
Loose (3.6Å)
Tight (3.4Å)
Corrected (3.5Å)
Elevation
Azimuth
# of images
E
GSFSC resolution cutoff at 0.143 (Å)
F
90°
G

Figure S5

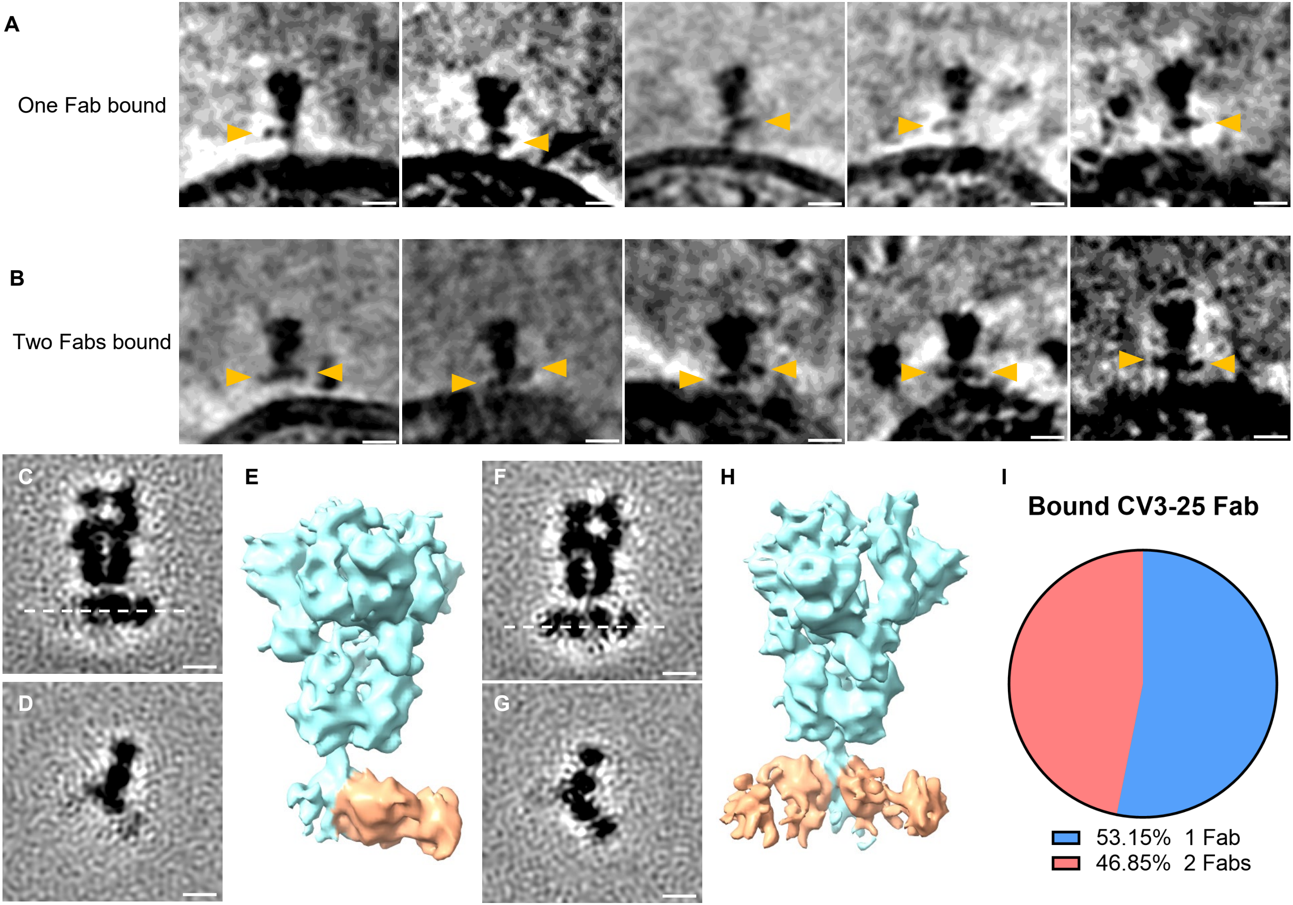
A
One Fab bound
B
Two Fabs bound
C
D
E
F
G
H
I
Bound CV3-25 Fab
53.15% 1 Fab
46.85% 2 Fabs

Figure S6

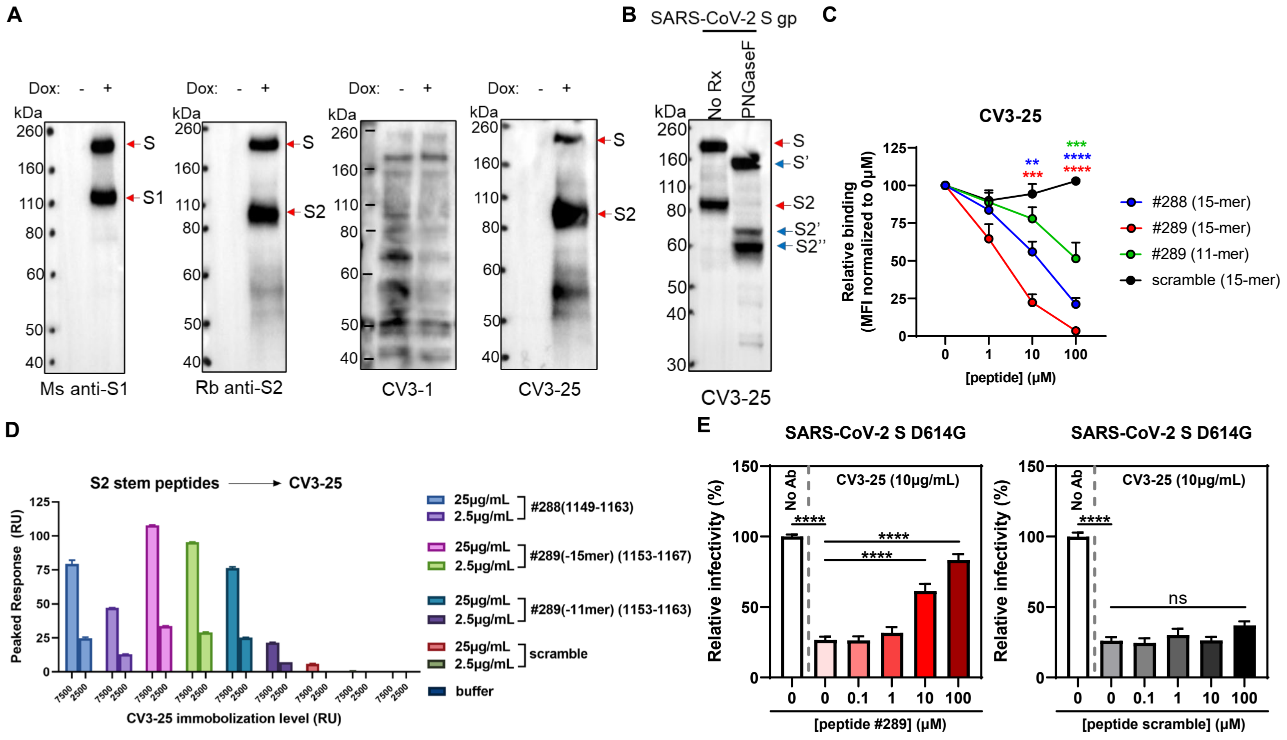

A
Dox: - +
kDa
260
160
110
80
60
50
40
S
S1
Ms anti-S1
S2
Rb anti-S2
CV3-1
CV3-25
B
SARS-CoV-2 S gp
No Rx
PNGaseF
S'
S2'
S2''
30
C
CV3-25
Relative binding (MFI normalized to 0μM)
[peptide] (μM)
#288 (15-mer)
#289 (15-mer)
#289 (11-mer)
scramble (15-mer)
D
S2 stem peptides → CV3-25
Peaked Response (RU)
CV3-25 immobolization level (RU)
25μg/mL
2.5μg/mL
#288(1149-1163)
#289(-15mer) (1153-1167)
#289(-11mer) (1153-1163)
scramble
buffer
E
SARS-CoV-2 S D614G
Relative infectivity (%)
No Ab
CV3-25 (10μg/mL)
[peptide #289] (μM)
[peptide scramble] (μM)
ns

Figure S7

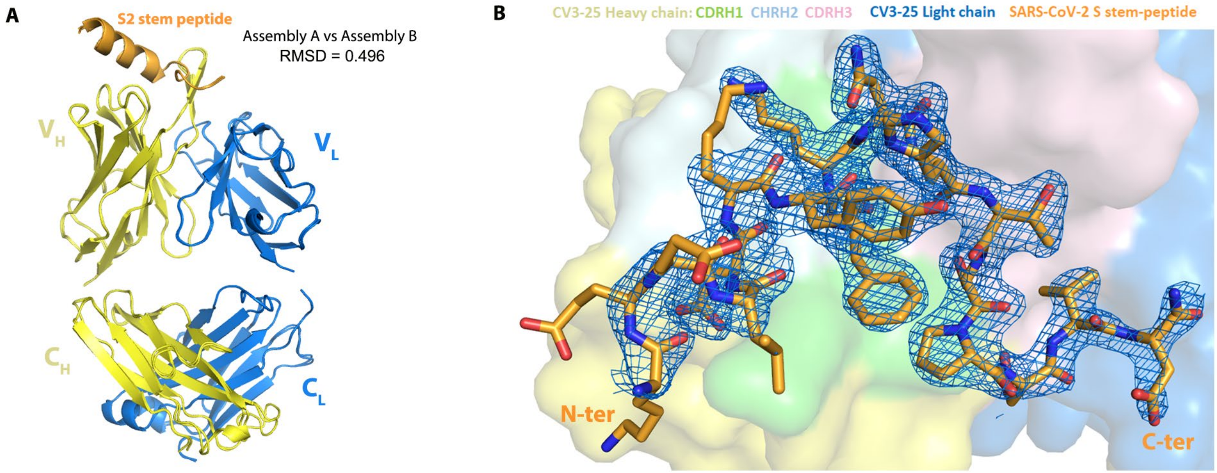

C

```
                     10        20        30          40        5052a     60        70        8082abc       90        100abcdefgh      110
                      |         |         |           |         | |       |         |          | |          |          |               |
                                          +  +++                  +  + +                                     +    + -
CV3-25_HV_mature     EVQLVESGAEVKKPGESLKISCKGSGYTFT RYWIG WVRQMPGKGLEWMG IIYPGDSDTRYSPSFQG HVTISADKSISTAYLQWNSLKASDTAMYYCAR LPQYCSNGVCQRWFDP WGQGTLVTVSS
CV3-25_HV_germline   EVQLVQSGAEVKKPGESLKISCKGSGYSFT SYWIG WVRQMPGKGLEWMG IIYPGDSDTRYSPSFQG QVTISADKSISTAYLQWSSLKASDTAMYYCAR LPQYCSNGVCQRWFDP WGQGTLVTVSS
CC40.8_HV            EVQLLESGGGLVQPGPSLRLSCAASGFTFS SYVHT WARQAPGKGLEWVS AIS-GTGYTYYADSVKG RFTVSRDNSKNTLFLQMSSLRAEDTAVYYCAI TMAPVV---------- WGQGTTVTVSS
B6_HV                EVQLQQSGPVLVKPGASVRMSCKASGYTIT DYYLN WVKQSHGKSLEWLG VLNPYSGGSLYSQTFKG KATLTVDRSSSTAYLELNSLTSEDSAVYYCAR QLGRGNGLDY------ WGQGTSVTVSS
                     -------------FWR1------------- CDRH1 -----FWR2----- ------CDRH2------ ---------------FWR3--------------- ------CDRH3----- ----FWR4---

                     10        20        29        30        40        50        60        70        80        90      96   100       107
                      |         |         |         |         |         |         |         |         |         |       |     |         |
CV3-25_LV_mature     EIVLTQSPSSVSASVGDRVTITC RASQGI------SSWLA WYQQKPGKAPKLLIY AASSLQS GVPSRFSGSGSGTDFTLTISSLQPEDFATYYC QQGNSFP--YT FGQGTNLEIK
CV3-25_LV_germline   DIQMTQSPSSVSASVGDRVTITC RASQGI------SSWLA WYQQKPGKAPKLLIY AASSLQS GVPSRFSGSGSGTDFTLTISSLQPEDFATYYC QQGNSFP--YT FGQGTNLEIK
CC40.8_LV            SYELTQPP-SVSVSPGQTARITC SGDALP------KRYAY WYQQKSGQAPILVIY EDKKRPS GIPERLSGSKSGTVATLTISGAQVEDEADYYC YSTDSSGNHAV FGGGTQLTVL
B6_LV                NIMMTQSPSSLAVSAGEKVTMSC KSSQSVLHSSDQKNYLA WYQQKPGQSPKLLIY WASTRES GVPDRFTGSGSGTDFTLTISSVQAEDLAVYFC HQYLSS---YT FGGGTKLEIK
                     ---------FWR1---------- ------CDRL1------ -----FWR2------ -CDRL2- --------------FWR3-------------- ---CDRL3--- ---FWR4----
```
